# The time course of fluid responsiveness

**DOI:** 10.1016/j.aicoj.2026.100036

**Published:** 2026-02-26

**Authors:** Ricardo Castro, Eduardo Kattan, Paulo Melo, Macarena Amthauer, Glenn Hernandez

**Affiliations:** a Departamento de Medicina Intensiva. Facultad de Medicina. Pontificia Universidad Catolica de Chile, Chile; b Intensive Care Unit, Hospital Clínico UC CHRISTUS, Santiago, Chile; c Intensive Care Unit, Hospital Santiago Oriente Dr. Luis Tisné Brousse, Santiago, Chile

**Keywords:** Fluid responsiveness

## Abstract

Fluid responsiveness (FR) is a cornerstone concept in the hemodynamic management of critically ill patients, referring to the heart's ability to increase output in response to fluid administration. This review explores the time course of FR, emphasizing its dynamic and transient nature influenced by various patient-specific factors and treatments. We highlight that FR is not a stable, binary state but rather a continuum that requires repeated evaluations using appropriate tools. We examine into factors affecting the temporal profile of FR, including the stage of resuscitation, fluid infusion rate, norepinephrine use, mechanical ventilation settings, systolic function, spontaneous breathing, and the patient's health condition. Understanding the duration and decay of the hemodynamic response is crucial for individualizing fluid therapy, potentially reducing the risks of both fluid overload and under-resuscitation. This review underscores the necessity of frequent reassessment of FR in critically ill patients and integrating this knowledge into personalized resuscitation protocols to improve clinical outcomes.

Fluid resuscitation remains a cornerstone of hemodynamic management in critically ill patients. Yet, the physiological response to fluid administration, quantified as fluid responsiveness (FR), is inherently transient and subject to multiple modulating factors. Although a substantial body of literature has focused on the diagnostic accuracy of tests to predict a positive FR state (FR+), less attention has been paid to the time-dependent behavior of this phenomenon.

Current understanding of FR tends to conceptualize it as a binary response to a single intervention, often assessed at a fixed time point. However, emerging evidence indicates that FR evolves dynamically over time, with both physiological mechanisms and clinical interventions influencing its persistence, resolution, or reappearance. Factors such as the type of fluid chosen, fluid infusion rate, vasopressor use, mechanical ventilation, and cardiac function play crucial roles in shaping the trajectory of FR over time. Recognizing these temporal dynamics is essential not only for optimizing fluid therapy but also for preventing iatrogenic harm, such as volume overload or inadequate perfusion.

This narrative review aims to discuss available data that characterize the time-related behavior of FR. Rather than evaluating the predictive performance of individual tests, our focus is on how FR status may evolve following a fluid challenge or preload-modifying maneuver and how this trajectory can inform clinical decision-making.

## Hemodynamic principles and preload dependency

Positive pressure ventilation can profoundly alter cardiovascular function [1]. This heart-lung interaction represents the interdependence between intrathoracic pressure (ITP), lung volume, and cardiovascular function [2]. Spontaneous inspiration decreases ITP, thereby lowering right atrial pressure (RAP) and increasing intra-abdominal pressure, which enhances venous return; however, excessive negative swings may collapse thoracic veins and limit flow [3,4]. Conversely, positive-pressure ventilation increases ITP, raising RAP and reducing venous return, while also decreasing left ventricular transmural pressure and left ventricle (LV) afterload [5,6]. These principles underpin functional hemodynamic monitoring, which utilizes cyclic ventilation as a standardized perturbation to assess FR [7,8]. Positive–pressure inspiration transiently reduces right ventricular preload and, subsequently, LV preload and output [2,9]. In patients with preserved biventricular reserve, this results in cyclic variations in stroke volume (SV) and pulse pressure, measurable as stroke volume variation (SVV) and pulse pressure variation (PPV). Both measures correlate proportionally with the degree of FR during controlled ventilation [10,11]. Conversely, if the heart is operating on the flat portion of the Frank–Starling curve, such variations are minimal or absent, and the patient is considered fluid unresponsive (FR−).

FR+ is currently defined as a ≥10–15% increase in cardiac output (CO) following a fluid challenge, typically using at least 4 mL/kg of crystalloid solution [12]. Static hemodynamic parameters, such as central venous pressure (CVP), have demonstrated limited predictive value, exhibiting performance no better than that of random chance [13]. In contrast, dynamic indices that leverage cardiopulmonary interactions, such as PPV and SVV, consistently demonstrate superior predictive accuracy, with areas under the curve (AUC) frequently exceeding 0.90 under controlled mechanical ventilation [14]. The passive leg raising (PLR) maneuver is a valuable tool for assessing these dynamic methods. By transiently increasing venous return, PLR acts as a reversible internal fluid challenge, enabling real-time assessment of FR without the risks associated with actual fluid infusion [15,16]. This approach allows clinicians to assess the potential hemodynamic benefits of volume expansion in a safe and physiologically informative manner.

The transient nature of FR+ is primarily driven by the rapid redistribution of fluid. The hemodynamic effects of both fluid boluses and PLR are inherently short-lived, as the administered volume or autotransfused blood quickly returns to peripheral or interstitial compartments [17,18]. Consequently, the temporal dynamics of FR+, encompassing both the initial rise and subsequent decline in CO, are modulated by a complex interplay of factors, including the phase of illness, type and rate of fluid administration, cardiovascular function, and endothelial integrity [19,20]. During early resuscitation, FR assessment informs whether preload augmentation improves CO and tissue perfusion [[21], [22], [23]]. In later phases, it may guide fluid removal in patients at risk of fluid overload or weaning failure [24,25]. Across all stages, FR represents an integrative marker of cardiovascular reserve, reflecting the heart’s capacity to adapt to hemodynamic stress [26].

Recent studies suggest that not only the presence but also the degree of FR+ may hold clinical relevance. The magnitude of CO increase among responders may vary [27], with smaller changes potentially exerting different clinical effects than more pronounced responses. Moreover, as the time course of FR is determined by the clinical setting and the assessment method [27,28], it must be interpreted accordingly.

## Factors that influence the time course of FR

1 Stage of fluid resuscitation

During the initial resuscitation phase, the goal of identifying FR+ is primarily to augment cardiac output (CO) and reverse acute circulatory failure. In contrast, during de-escalation, once hemodynamic stability is achieved, restoring or maintaining a FR+ state may help guide fluid removal. These divergent clinical goals may influence both the magnitude and temporal trajectory of FR, as developed in the following sections.

Importantly, the time course of FR may be strongly influenced by the clinical setting, the method of assessment, and the underlying physiological context, each of which introduces variability in detection and trajectory. Among these contextual factors, fluid tolerance [29] plays a secondary yet clinically relevant role. For example, in early sepsis with increased endothelial permeability, the interplay between intravascular depletion and interstitial redistribution can produce transient FR+ states even in the presence of low fluid tolerance. Notably, venous congestion signals, often markers of fluid intolerance, can coexist with FR+ early during ICU admission [30], potentially modulating the duration FR+.

Fluid therapy, as conceptualized in the ROSE model (Resuscitation, Optimization, Stabilization, Evacuation) [29], should be viewed as a continuum rather than as distinct, sequential compartments. In this model, de-escalation spans both the Stabilization and Evacuation phases, emphasizing progressive efforts to prevent or reverse fluid accumulation. Transitions in FR status within and across these phases reflect dynamic physiological adaptation and evolving therapeutic goals. For this review, we define resuscitation as the period of active fluid administration, and de-resuscitation as the phase during which active fluid removal is pursued.

### Resuscitation phase

Two decades ago, Michard and Teboul reported a FR+ rate of 52% based on a pooled analysis of 12 clinical studies involving 334 patients [31]. More recently, Messina et al. [32] confirmed a similar estimate (53% of FR+ at baseline) in a meta-analysis of 124 studies involving 6,086 patients, most of whom had sepsis or septic shock. However, these aggregate data reflect a static snapshot. Addressing the temporal dimension, Roger et al. [28] administered 500 mL crystalloid bolus over 10 min to septic shock patients and found that 53% were responsive at 10 min (T10), defined by a >15% increase in SV. Yet, only 51.3% of these remained FR+ at 30 min (T30), while 6% of initial FR− became FR+ by T30. Importantly, classification at T30 should be interpreted cautiously, as no new functional assessment was performed.

**Table 1:** Determinants of the time course of fluid responsiveness in critically ill patients.

Determinant	Effect on FR Time Course	Clinical Implication	Key References
Phase of resuscitation	Acute: 50% FR+ at admission; frequent shift to FR- over time Deresuscitation: fluid removal prolongs FR+ duration.	Acute: early redistribution in hypermeability conditions shortens intravascular expansion and duration of FR+ Deresuscitation: endpoint determines FR time course. Fluid removal does not ensure FR+.	Messina (2022) Kattan (2020) Castro (2024)
Infusion rate	Rapid boluses: larger but short-lived responses. Slow infusions may obscure FR+ status due to ongoing redistribution.	Use rapid infusions when an immediate volume-expanding effect is desired.	Toscani (2017) Kattan (2020)
Vasopressor use	NE increases stressed volume and shortens FR+ duration. Higher doses may mask FR+ Dose changes shift vascular tone and modulate FR stat. us	Interpret FR the in context of NE titration history. Lowering NE dose may induce FR+ state.	Persichini (2012) Monnet (2016)
Mechanical Ventilation	High Vt amplifies cardiopulmonary interaction detectability. Low Vt may shorten detectable FR+ duration. PEEP exerts a context-dependent modulation extending or shortening the time course of FR	Use tidal volume challenge or adapted dynamic tests. Always consider lung recruitability and RV function when assessing PEEP effects on FR.	De Backer (2005) Myatra (2017) Wang (2023) Jellinek (2000) Xingzheng (2024) Borlino (2024)
Cardiac function	Impaired contractility blunts SV response to preload changes	Tailor FR interpretation to contractile reserve, volume status and chronotropic compensation.	Sarnoff (1954) Michard (2003) Si (2018)
Spontaneous breathing	Irregular ITP changes; PPV unreliable; PLR remains usable. Vt challenge enhances cardiopulmonary interactions, improving PPV’s predictive accuracy	Prefer PLR or UBE. Vt challenge may improve PPV accuracy, though FR+ duration remains poorly defined.	Hamzaoui (2021) Kim (2024)
Special conditions	AF reduces SV consistency, limiting use of PPV and SVV; FR course is unpredictable in sepsis.	Avoid PPV-based tools in AF. Use serial PLRs. Expect shorter FR+ in septic conditions.	Kim (2016)

FR+, positive fluid responsiveness.

FR−, fluid unresponsiveness.

NE, norepinephrine.

Vt, tidal volume.

SV, stroke vokume.

ITP, intrathoracic pressure.

UBE, upper body elevation.

PPV, pulse pressure variation.

PLR, passive leg raising.

AF, atrial fibrillation.

SVV, stroke volume variation.

**Fig. 1:**
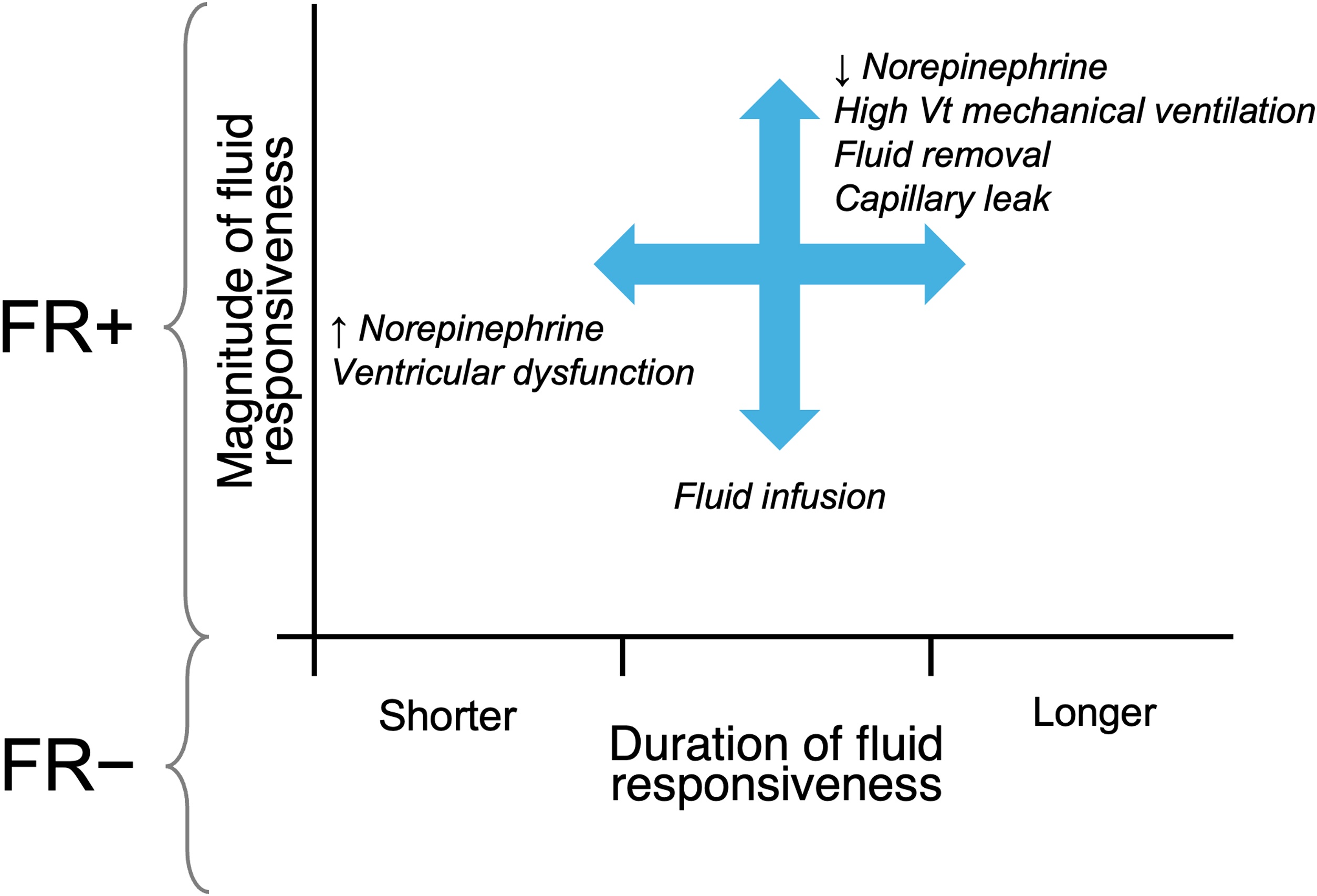
Evolution of fluid responsiveness during protocolized resuscitation, according to fluid responsiveness state at baseline.

At a larger scale, ANDROMEDA-SHOCK [32] was the first major study to incorporate a systematic per-protocol assessment of FR in patients with early septic shock. In this study, 57% of patients were fluid responsive at the time of recruitment, with no significant differences between the peripheral perfusion-targeted and lactate-targeted resuscitation groups. Of note, this assessment was done after initial fluid loading (at least 20 mL/kg). During the 8 -h intervention period, most patients transitioned into FR− [33]. Notably, <15% of patients initially classified as FR− became FR+ at any time. Additionally, only 13 patients who were FR+ at baseline maintained this status by the end of the intervention (Fig. 1). The study found that 30% of patients were already FR− before starting ICU-based resuscitation, and these FR trajectories impacted therapy. In fact, FR− patients received significantly less fluid compared to FR+ patients, with around 1000 mL less during the first 2 h and 1500 mL less over the entire 8 -h period. However, despite the changes in FR status, both FR+ and FR− patients achieved comparable resuscitation targets and exhibited similar outcomes in terms of mortality and improvement in organ dysfunction. Incidentally, this finding suggests that the time course of FR does not necessarily correlate with adverse clinical outcomes when fluid administration is appropriately managed.

**Fig. 2:**
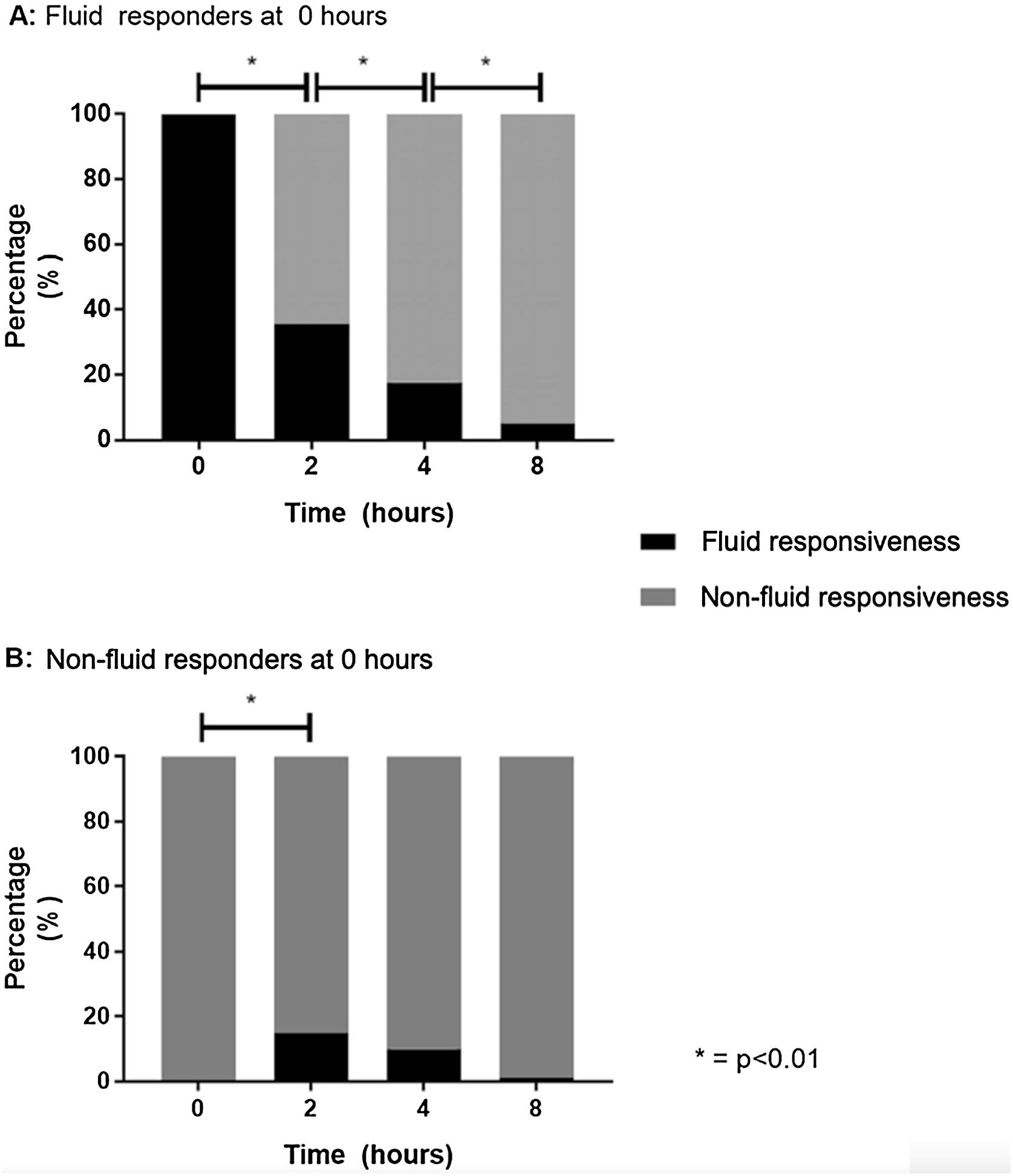
Illustration on the temporal profile of FR. The X-axis represents time after fluid challenge (e.g., T0, T10, T30, T60 min), and the Y-axis represents the percentage increase in SV or CO. The graph shows the initial increase in the hemodynamic response immediately following fluid administration, followed by a progressive decline due to rapid fluid redistribution. The arrows denote the influence of key modulating factors on the magnitude and duration of the response.

The decline in CO observed in patients from these studies [28,34] is plausibly explained by fluid administration and early intravascular redistribution [35]. In a minority of patients, the delayed increase in SV may reflect subsequent recruitment of preload reserve through norepinephrine-driven redistribution of blood from unstressed to stressed vascular compartments [36] and/or improvement in ventriculo-arterial coupling [37]. Measurement variability in echocardiographic SV assessment, as acknowledged by Roger et al., is also likely to contribute to some of these transitions.

These observations underscore the highly dynamic, sometimes unpredictable time course of FR during the acute phase of septic shock. Moreover, early signs of fluid intolerance [29,30,38] in FR+ and still hypoperfused patients may paradoxically prolong FR+ by prompting clinicians to appropriately restrict further fluid administration. This creates a challenging subset of persistently hypoperfused yet FR+ individuals in whom additional fluid would be physiologically effective but clinically hazardous.

### De-escalation phase

In a study by Castro et al. [39], 67% of patients diagnosed with fluid overload were FR+ at baseline before any depletive intervention. When fluid removal was titrated with the explicit goal of achieving FR+, all patients reached an FR+ state within 12 h. In contrast, an empirically guided strategy targeting only negative fluid balance left 42% of patients FR− even after 40 h. In this approach, FR+ served not only as a surrogate for preload correction but also as a safety endpoint to guide fluid removal. This strategy was particularly intended to secure weaning from mechanical ventilation, where persistent FR− has been associated with adverse outcomes such as pulmonary edema and extubation failure [40]. One more time, fluid tolerance may further modulate the FR time course, as patients with fluid overload and lower tolerance may exhibit prolonged FR− phases, making prediction and management more challenging during transitions. Monitoring for venous congestion alongside FR can help navigate these overlaps, as FR+ with fluid intolerance states may persist or emerge, requiring tailored de-escalation to mitigate risks like organ dysfunction [30,41]. A complementary perspective was provided by the randomized trial conducted by Bitker et al. [42]. In this study, patients initially receiving continuous norepinephrine infusion for acute circulatory failure underwent fluid removal guided by a structured hemodynamic protocol, which incorporated serial assessments of FR and tissue perfusion. FR was evaluated every 4 h using PLR or Trendelenburg positioning, and ultrafiltration rates were adjusted accordingly. FR+ was observed in only 26% (84/327) of assessments over a 72-h period. Importantly, FR+ functioned not only as a marker of preload reserve but also as a threshold to attenuate or suspend ultrafiltration, aiming to prevent hemodynamic instability.

Together, these studies underscore a bidirectional interaction: fluid removal may promote the emergence and persistence of FR+, whereas the appearance of FR+ subsequently limits the extent of ongoing fluid extraction. In fact, FR+ has been associated with new episodes of hypotension during fluid removal [43], and FR status may serve as a warning signal. This dynamic feedback illustrates how clinical objectives and physiological responses jointly shape the trajectory of FR during the de-escalation phase. It also underscores the importance of frequent FR reassessment during this period, when the risks of fluid overload increase and ventilator weaning approaches. These findings reinforce that FR phases are not discrete but instead reflect a continuum influenced by individual patient factors and clinical contexts, such as the early coexistence of FR+ and venous congestion, within multi-phase frameworks like ROSE [30].

2 Type of fluid

The physicochemical properties of the administered fluid are a fundamental determinant of the temporal trajectory of FR. While the magnitude of the initial CO increase depends largely on the patient’s preload reserve or Frank-Starling status, the duration of this effect is determined by the fluid’s volume kinetics.

Crystalloid solutions, which lack oncotic pressure, rapidly redistribute from the intravascular to the interstitial compartment. Mathematical modeling of volume kinetics demonstrates that the intravascular half-life of crystalloids in conscious volunteers is short, approximately 20−30 min [44]. Consequently, the FR− state induced by a crystalloid bolus is inherently transient, often dissipating before the clinical infusion is even completed if the rate is slow. In contrast, colloids maintain intravascular volume expansion for significantly longer periods due to their oncotic properties, resulting in a probable flatter decay curve and a shorter FR+ status [45].

This distinction is particularly relevant in sepsis, where shedding of the glycocalyx, a key regulator of vascular permeability, is the specific pathological mechanism that accelerates the transition from stressed to unstressed volume or interstitial edema [46,47]; in consequence, the FR+ time course may be longer in septic shock compared to, for example, hypovolemia from hemorrhage or dehydration, because the barrier that retains fluid within vessels is compromised. Therefore, the clinician must anticipate that a patient may transition from FR+ to FR− much more rapidly after a colloid challenge than after a crystalloid challenge, not because cardiac function has changed, but because the preload boost may dissipate more rapidly depending on the type of fluid administered.

3 Fluid infusion rate

In addition to the type of fluid, the infusion rate directly modulates the time-dependent dynamics of FR status. A randomized controlled trial demonstrated that the proportion of FR+ patients declined from 51.0% when a 4 mL/kg bolus was infused over 10 min to 28.5% when the same volume was administered over 20 min [48]. However, this study was conducted in a small cohort of neurosurgical patients under stable hemodynamic conditions, which limits the generalizability of its findings. Consistent results were reported in a meta-analysis, where fluid responsiveness was significantly less frequent when fluid challenges were administered over ≥30 min (49.9%) compared to <15 min (59.2%, p = 0.04). Notably, no difference was found between <15 and 15–30 min infusions (57.7%, p = 1.0) [49].

These findings are physiologically plausible. Rapid fluid administration induces an acute increase in intravascular volume and venous return before compensatory mechanisms such as baroreflex-mediated sympathetic activation, vasoconstriction, and fluid redistribution can attenuate the hemodynamic effect [50]. In contrast, slower infusions may allow these mechanisms to engage during the infusion itself, potentially blunting the observed rise in SV or CO. This complicates the prediction of FR and highlights the importance of time-sensitive assessments, particularly in dynamic clinical settings.

Moreover, infusion rate may be especially relevant in the evolution of FR in patients with capillary leak syndrome, where crystalloids rapidly extravasate into the interstitial space, reducing their effective intravascular volume expansion [51]. While our review does not compare specific fluid challenge protocols, it is worth noting that techniques such as the mini-fluid challenge (e.g., 100 mL over 1 min) have been shown to reliably predict FR+ while minimizing the risk of fluid overload and eventual interstitial leakage [52].

Although not intended to assess the time course of FR, the mini-fluid challenge avoids unnecessary additional fluid administration and may reduce fluid loading as a confounding factor when interpreting temporal changes in FR. However, technical limitations of the monitoring device must be considered, particularly when using mini-fluid challenges; long averaging windows (e.g., 60 seconds) may smooth out the transient peak of a rapid fluid response, potentially leading to false negatives FR assessments if the monitor cannot track real-time changes [53].

4 Vasopressor use

Vasopressors modulate both vascular tone and stressed volume, exerting a continuous vasoactive influence that contrasts with the transient stimuli used to assess FR, such as fluid boluses or passive leg raising. This ongoing physiological modulation introduces a dynamic backdrop against which FR must be interpreted.

Norepinephrine is the most extensively studied vasopressor in this context. As a catecholamine, it acts primarily via α-adrenergic receptors to induce arterial and venous vasoconstriction while also stimulating β-receptors, thereby exerting inotropic, plus other effects [54,55]. It is the first-line vasopressor in septic shock [56], primarily used to restore the mean arterial pressure (MAP) and optimize organ perfusion. However, its effects also influence the magnitude and duration of the hemodynamic response to fluid administration. Norepinephrine increases venous return by constricting venous capacitance vessels [57] and elevating the mean systemic filling pressure (MSFP) [58]. This enhances preload and the heart’s operating point on the Frank–Starling curve, potentially amplifying the magnitude and duration of SV and CO responses to fluid administration [58]. Its impact on FR is dose-dependent and context-specific. An important caveat is that reducing norepinephrine dosage may shift the hemodynamic state from FR− to FR+ by secondarily increasing venous compliance and derecruiting stressed volume. This mechanism must also be considered when interpreting the duration of FR if norepinephrine dosing has been adjusted [59].

Although less extensively studied, vasopressin may influence FR through mechanisms involving V1a receptor stimulation, which increases systemic vascular resistance and afterload [60,61]. However, experimental data from animal studies suggest that vasopressin could also increase whole-body vascular capacitance via baroreflex-mediated sympathetic withdrawal, potentially reducing stressed volume and MSFP [62]. These findings, however, come from healthy animal models and may not extrapolate directly to septic patients. In fact, in clinical shock states, vasopressin is primarily recognized as a potent vasoconstrictor, and its net effect on MSFP is uncertain, potentially increasing venous tone but also resistance to venous return. As such, its direct contribution to FR remains uncertain and likely varies by context. Additionally, via V2 receptor-mediated antidiuresis, vasopressin may lead to gradual intravascular volume expansion, producing a slow endogenous fluid load, progressively increasing preload and shifting the heart's position along the Frank–Starling curve, thereby diminishing FR+ without further fluid administration. Although speculative, such a mechanism may be relevant in the post-acute phase of septic shock, where vascular integrity is restored, and water retention remains intravascular [33]. This underscores the dynamic and context-dependent impact of vasopressin on FR status.

5 Mechanical ventilation

Mechanical ventilation parameters, particularly tidal volume (Vt) and positive end-expiratory pressure (PEEP), influence cardiopulmonary interactions and shape dynamic cardiovascular indices such as pulse pressure variation (PPV) and stroke volume variation (SVV). Although no study has directly quantified how ventilator settings alter the temporal profile of FR, the established linear relationship between Vt, pulmonary driving pressure, transpulmonary pressure, and right ventricular afterload [63], suggests that both Vt and PEEP influence the presence, magnitude, and duration of FR through their effects on preload and cardiac function.

Higher Vt increases intrathoracic pressure swings and augments cyclic preload variation, improving the discriminative performance of PPV. De Backer et al. [64] demonstrated that PPV reliably predicts FR+ in mechanically ventilated patients only when Vt is ≥8 mL/kg. Similarly, Biais et al. [65] showed that PPV performance improves when Vt is ≥8 mL/kg and driving pressure exceeds 20 cmH₂O. These findings illustrate that Vt influences both the magnitude of dynamic indices and, by extension, the clinical window during which FR+ can be detected and acted upon.

Conversely, low-Vt ventilation (<6 mL/kg), as employed in lung-protective strategies, reduces intrathoracic pressure variability and may obscure otherwise present FR+, particularly when relying on PPV, shortening a FR+ period [66]. This may be highly relevant in the presence of hypoperfusion that demands an increase in oxygen transport via CO augmentation with fluids. In such settings, a Vt challenge can unmask FR+ by transiently restoring sufficient preload variation [67].

PEEP modifies right ventricular preload and afterload by increasing intrathoracic pressure, raising right atrial pressure, and altering the pressure gradient with MSFP. Depending on the clinical context and alveolar recruitability [68,69], PEEP may reduce venous return [1,70], leave it unchanged [71,72], or even increase it [72]. These context-dependent effects can extend or shorten the observed FR+ state. Elevated PEEP may accelerate transition to FR− by progressively limiting venous return, or conversely may prolong apparent FR+ in patients with right ventricular dysfunction by progressively impeding venous return over time [73,74]. Clinicians must therefore remain cognizant that ventilatory adjustments may influence the evolving trajectory of FR when interpreting dynamic indices and timing fluid interventions.

Overall, mechanical ventilation parameters are integral to FR prediction tools. Their relevance lies not in prescriptive application, but in demonstrating how physiologic interactions between ventilation and venous return contribute to variability in the clinical and temporal expression of FR.

6 Cardiac function

Experimental studies in animal models, subsequently confirmed in humans, demonstrated that inotropy and afterload significantly modulate the preload–CO relationship [73,74]. In normal hearts, which typically operate along the ascending portion of the Frank–Starling curve, SV increased steeply in response to preload augmentation. In animal models, epinephrine shifted the Frank-Starling curve upward and to the left, enhancing preload responsiveness. Notably, this FR+ effect persisted for up to 12 min after discontinuation of norepinephrine infusion [73], but no further assessments were performed. In contrast, patients with heart failure, operating at higher filling pressures, exhibited a blunted SV response, reflected in a flattened curve and reduced contractile reserve. These findings primarily manifest acute hemodynamic behaviors, and the temporal evolution of this effect was not systematically characterized.

It was established that the slope of the relationship between preload and SV depends mainly on ventricular contractility [75]. However, Michard et al. [76] demonstrated that, in septic shock patients, baseline preload modulated the ventricular response to fluid challenges regardless of systolic function. As the study focused on acute hemodynamic responses, the temporal evolution of these effects was not assessed. Importantly, during dynamic testing, CO reflects both SV and heart rate. In consequence, reflex-mediated chronotropic adaptations (e.g., bradycardia) may obscure significant changes in SV [77,78]. Ultimately, although systolic function and preload status shape the initial response to fluid loading, the duration and decay of this effect remain insufficiently characterized in both experimental and clinical contexts. Prospective studies are needed to clarify how systolic performance governs the persistence of FR+ over time.

7 Spontaneous breathing

Assessing FR in spontaneously breathing patients is challenging, as traditional dynamic indices like PPV and SVV are less reliable in this context [79] and even less informative regarding the duration of FR. In non-ventilated patients, inspiration decreases ITP, increasing venous return and SV in preload-dependent states, while expiration reverses this effect [80]. Recent studies support PLR and echocardiography maneuvers as preferable alternatives [81,82]. However, the inclusion of both ventilated and non-ventilated patients, as well as the absence of time-resolved data on SV, CO, or PPV changes, has limited the characterization of the FR trajectory in this group.

The Vt challenge enhances cardiopulmonary interactions and has been shown to improve the predictive performance of PPV in patients with assisted or spontaneous breathing [79]. Additionally, Kim et al. [83] demonstrated that a decrease in SV of ≥11.8% during 60 ° upper body elevation predicted FR accurately, without the need for fluid administration; however, the duration of this response was not assessed. Similarly, Reich et al. [84] and Terai et al. [85] reported that both Trendelenburg and PLR increased CO within one minute in ventilated and spontaneously breathing subjects. Although PLR, according to the authors, appeared to sustain its hemodynamic effect for a longer time, the findings are limited by small sample sizes and heterogeneous populations.

Altogether, these considerations underscore the lack of information regarding the time course of FR in this setting, the physiological limitations of the Trendelenburg position, the need to distinguish between ventilation modes, and the importance of generating more granular temporal data when evaluating postural maneuvers for FR in spontaneously breathing patients.

8 Special considerations

The assessment of FR is particularly challenging in patients with non–sinus rhythms, notably atrial fibrillation (AF), the most common arrhythmia in critically ill populations [86]. AF generates intrinsic beat-to-beat variability in SV, irrespective of ventilatory mode, thus compromising the reliability of dynamic preload indices such as PPV and SVV. This variability also confounds the interpretation of hemodynamic responses to fluid challenges or postural maneuvers. Although extended automated averaging windows may mitigate AF-related signal noise, the overall diagnostic accuracy of FR assessment in this context remains uncertain. For example, in a study of 43 post-cardiac-surgery patients with AF, Kim et al. [87] reported that a ≥7.3% increase in SVI during PLR predicted FR+ (sensitivity, 71%; specificity, 79%). While this suggests that preload-modifying maneuvers may retain diagnostic utility, it also underscores substantial diagnostic imprecision and the continued influence of arrhythmia-related variability.

These limitations are further compounded when attempting to characterize the temporal evolution of FR in patients with AF. Given the uncertainty in identifying FR+ at a single time point, assessing its behavior over time is even more challenging and remains unstudied under standardized conditions. Despite their clinical relevance and high prevalence, patients with AF are notably absent from current empirical frameworks on the time course of FR, representing a critical area for future research.

Additionally, intra-abdominal hypertension (IAH) represents a mechanical barrier to venous return. High intra-abdominal pressure acts as a resistor, blunting the hemodynamic response to PLR and creating a false FR− state, thereby rendering the maneuver's results less reliable [88].

Easily obtainable bedside measures, such as capillary refill time (CRT), could have a role in tracking the temporal evolution of FR. In a study by Raia et al. [89], CRT was shown to reflect the transient hemodynamic improvement following PLR-induced autotransfusion, suggesting potential clinical utility. However, CRT responses to CO increase interventions are not consistently predictable [90]. Whether CRT trajectories can reliably mirror the time course of FR across different clinical contexts remains uncertain and warrants further investigation.

## Summary and conclusion

FR is a dynamic state that is dependent on patient–specific and treatment–related factors. In septic shock, particularly in the acute phase, it remains highly variable and often unpredictable. Over time, most acute patients transition to a FR− state, necessitating adjustments in fluid administration strategies. The important lesson is that FR should not be assessed once and assumed stable. Instead, repeated evaluations with appropriate tools are required to track its time course and interactions with other physiological variables. Given the multiple factors that affect its temporal profile, frequent reassessment is necessary.

Furthermore, FR is not a binary state but a continuum, where the magnitude of response informs tailored management strategies. Quantifying the duration and decay of the hemodynamic response enables individualized resuscitation and de-escalation approaches, potentially reducing the risks of both fluid overload and under-resuscitation. A deeper understanding of these dynamics is fundamental to optimizing and personalizing resuscitation in critically ill patients, with the potential to improve clinically relevant outcomes.

In conclusion, FR is a dynamic state requiring continuous evaluation in critically ill patients. Its rapid evolution underscores the necessity of frequent reassessment using reliable dynamic indices. Understanding the time course of FR and integrating this knowledge into personalized resuscitation protocols may improve clinical outcomes in patients with septic shock and other critical conditions.

## Declaration of competing interest

No competing interest.
